# Identification and characterization of a novel molecular classification based on disulfidptosis-related genes to predict prognosis and immunotherapy efficacy in hepatocellular carcinoma

**DOI:** 10.18632/aging.204809

**Published:** 2023-07-03

**Authors:** Li Yang, Weigang Zhang, Yifeng Yan

**Affiliations:** 1 Department of Forensic Pathology, Wannan Medical College, Wuhu, China; 2 Department of Graduate School, Wannan Medical College, Wuhu, China

**Keywords:** hepatocellular carcinoma, disulfidptosis, tumor microenvironment, immunotherapy, WGCNA

## Abstract

Background: Disulfidptosis has been discovered as a mechanism of cell death mediating by SLC7A11. Nonetheless, little is known about the relationship between disulfidptosis-related genes (DRG) and hepatocellular carcinoma (HCC).

Methods: 7 datasets including 1,302 HCC patients and 62,530 cells were downloaded. We adopted consensus clustering algorithm to construct the consensus matrix and cluster the samples’ DRG related expression profile data. Then, weighted gene co-expression network analysis (WGCNA) was conducted to identify hub gene modules associated with the identified clusters and determine the correlation between modules. A DRG.score was constructed based on genes through differential analysis and WGCNA of the 2 clusters.

Results: Univariate and multivariate Cox regression analysis show that SLC7A11 and LRPPRC can be used as an independent factor in HCC. Then, two molecular subgroups with significantly different survival were identified based on 10 DRG. The cluster.A showed a worse prognosis, higher immune infiltration, and higher immune checkpoint expression. Then, by differential analysis and WGCNA of the 2 clusters, we identified 5 hub genes, and constructed a DRG.score. Univariate and multivariate Cox regression analysis show that DRG.score can be used as an independent factor to predict the prognosis in HCC. Furthermore, high DRG.score group had a worse prognosis, and was validated in TCGA-LIHC, LIRI-JP, GSE14520, GSE36376, and GSE76427. Preclinically, patients with higher DRG.score demonstrated significant immunotherapy therapeutic advantages and transcatheter arterial chemoembolization clinical benefits.

Conclusions: SLC7A11 and LRPPRC play an essential role in HCC prognosis prediction. The DRG.score might become useful biomarkers for novel therapeutic targets.

## INTRODUCTION

Hepatocellular carcinoma (HCC) is a common malignant tumor of the digestive system. HCC cells grow rapidly and are characterized by high vascular invasion and metastasis inside and outside the liver, resulting in poor treatment for HCC patients, with a 5-year survival rate of only about 15%, making it the fifth most common cancer in the world [1]. Despite aggressive surgical resection, radiofrequency ablation, transcatheter arterial chemoembolization, and chemotherapy, most patients still die from tumor metastasis and recurrence [2, 3]. The problems of poor prognosis and high drug resistance have been difficult to solve. The current availability of immune checkpoint inhibitors (ICIs) therapies for the treatment of a variety of malignancies suggests that ICIs may open up new avenues for the clinical management of HCC [4, 5]. However, no predictive biomarkers for the therapeutic efficacy of ICIs have been constructed in HCC. Therefore, the search for effective biomarkers of ICIs in HCC is particularly critical.

Early diagnosis and subsequent treatment are an urgent problem in medicine, and the elucidation of the proliferation and invasion and metastasis-related proteins and signaling pathways of HCC is one of the current hot spots. With the continuous development of basic medicine and clinical medical technology, researchers have found that the development of HCC is closely related to the dysregulation of cell death [6, 7]. Abnormal accumulation of intracellular disulfides in SLC7A11 high cells under glucose starvation conditions induces cell death called disulfidptosis [8]. Despite the success of chemotherapy in clinical cancer treatment, resistance to chemotherapeutic agents caused by genetic mutations remains a challenge. Disulfidptosis is gradually being recognized as a new therapeutic pathway for the elimination of malignant cells, and it plays a key role in suppressing tumorigenesis, especially in tumors that are resistant to conventional chemotherapy.

In this work, our first step was to assess the different expression of disulfidptosis-related genes (DRG) in HCC and normal tissues. Subsequently, we used multiple bioinformatics approaches to comprehensively assess the association of DRG expression with HCC clinicopathology and prognosis. Then, we comprehensively studied the accuracy of the model based on DRG for the prognosis, clinical characteristics, and ROC of HCC patients in the training and validation sets, and analyzed the enrichment pathway of HCC patients in the high-low risk group.

## MATERIALS AND METHODS

### Data acquisition

The mRNA expression data of HCC were further retrieved by searching the GEO database with the following keywords: “hepatocellular carcinoma”, “HCC”, and “liver cancer”. After initial screening, 3 gene expression omnibus (GEO) profiles with clinical information (GSE14520 [9], GSE36376 [10], and GSE76427 [11]) were selected and downloaded. The profiles base on GPL571, GPL10558, and GPL10558 platform. 221 samples in GSE14520, 223 samples in GSE36376, 115 samples in GSE76427. We also adopted ICGC (tumor: 231) and TCGA (tumor: 365; normal: 50) public database to download the RNAseq data. A total of 1,155 HCC samples with clinical information were selected. We also downloaded transcatheter arterial chemoembolization (TACE) dataset (GSE104580) to predict the clinical value of DRG.score, and a single-cell RNA dataset (GSE140228) was downloaded to explore the expression of 2 hub genes (Table 1) [12].

**Table 1:** The details of 7 datasets enrolled in this study.

**Accession number**	**Platform**	**Number of cells/patients**	**Treatment information**
TCGA-LIHC	Illumina RNAseq	365	No
ICGC-LIRI-JP	Illumina RNAseq	231	No
GSE14520	Affymetrix Human Genome U133A Array	221	No
GSE36376	Illumina HumanHT-12 V4.0 expression beadchip	223	No
GSE76427	Illumina HumanHT-12 V4.0 expression beadchip	115	No
GSE104580	Affymetrix Human Genome U133 Plus 2.0 Array	147	TACE Treatment
GSE140228	Illumina HiSeq 4000	62,530 cells	No

### Data preprocessing

A FPKM gene expression matrix was acquired from TCGA and converted into TPM format [13]. The merged expression matrix was then eliminated from batch effects and normalized using the R package “sva” [14].

### Analysis of differential DRG in TCGA-LIHC

The DRG expression was extracted from the collated RNA sequence data. DEG of DRG were analyzed in HCC tissues and normal tissues using the “limma” package [15].

### Cox univariate and multivariate analysis for DRG in TCGA-LIHC

The “Survival” data package was used to analyze the differential genes by Cox single factor analysis, and the hazard ratio (HR) and *P* value were obtained. The intersect genes with P < 0.05 in two survival analyses were selected by R software. Gender, age, TNM staging, AFP and DRG were subjected to univariate and multivariate COX regression analyses. The relationship between clinicopathological characteristics and DRG and survival prognosis of patients with HCC was investigated. The sensitivity and specificity of DRG and different clinical characteristics in predicting survival prognosis of HCC patients were assessed by the area under the curve, and the predictive ability of different clinicopathological characteristics and DRG was compared.

### Molecular subtypes base on 10 DRG in meta cohort

We inferred consensus cluster based on the DRG expression using the R package ConsensusClusterPlus. The optimal cluster number k was chosen depending on the elbow and CDF curve [16].

### Immune cell infiltration analysis

ESTIMATE was utilized to reveal TME in tumor tissues [17]. MCP counter, ssGSEA, cibersort (https://cibersortx.stanford.edu/), EPIC, and TIMER were utilized to reveal the immune cell infiltration [18–20].

### Differential gene analysis between the 2 clusters and GO and KEGG enrichment analysis

“Limma” R package was performed to detect putative differences between 2 clusters (|log2FC|>1; adj. p<0.05) [21, 22].

### WGCNA

The R language “WGCNA” package was used to construct a gene co-expression network for the normalized gene data and determine the optimal soft threshold. A scale-free network is constructed based on the optimal soft threshold, and the genes are subsequently clustered into the dynamic tree cutting algorithm is used to cluster and classify the genes into different color functional modules. Gene significance (GS) indicates the correlation between genes and traits, while module membership (MM) indicates the correlation between module feature vectors and gene expression profiles. The correlation between modules and clinical traits was analyzed by Pearson algorithm by combining GS and MM, and the module with the highest correlation with clinical traits of HCC was selected as the key module in this study [23].

### DRG.score model construction

Based on the DEGs and WGCNA of the 2 clusters, we established a DRG.score by using principal component analysis (PCA) method. The DRG.score of each sample was calculated by


$$\text{DRG}\text{.score}=\sum (P\text{C}1_{i}+P\text{C}2_{i}),$$

where “i” represents the gene expression level of hub gene expression.

### GSEA

GSEA judges the enrichment of gene sets based on gene expression. The difference between the two lies in that GSEA judges the enrichment of gene sets based on the contribution of genes in gene sets. To assess the signaling pathways associated with the prognostic model, we used GSEA to assess enrichment pathways in the high-risk and low-risk groups [24].

### Immunotherapy efficacy analysis

Use the TIDE web server to predict each sample’s response to immunotherapy based on liver cancer transcriptomic data. Individual TIDE scores were pooled to predict the efficacy of treatment with ICIs in high- and low-DRG.score groups, with higher TIDE scores indicating that tumor cells are more prone to immune escape, implying a lower response rate to ICIs treatment [25].

### Statistical analysis

Correlations between variables were explored using Spearman or Pearson coefficients. Continuous variables that conformed to the normal distribution were compared using independent t-tests for comparisons between binary groups, while continuous variables with skewed distributions were compared with the Mann–Whitney U test. Survival curves for categorical variable prognostic analyses were generated using the Kaplan–Meier method, while the log-rank test was used to estimate statistical significance. The significance level was set at P < 0.05, and all statistical tests were two-sided. All statistical data analyses were performed using R software or online analysis tools described in the relevant Materials and Methods subsections.

### Data availability

All data used in the study can be downloaded from the TCGA data repository (https://portal.gdc.cancer.gov/repository), ICGC data (https://icgc.org/) and the GEO database (https://www.ncbi.nlm.nih.gov/gds/?term=).

## RESULTS

### mRNA expression levels and predictive efficiency of 10 DRG in TCGA-LIHC cohort

**Figure 1:**
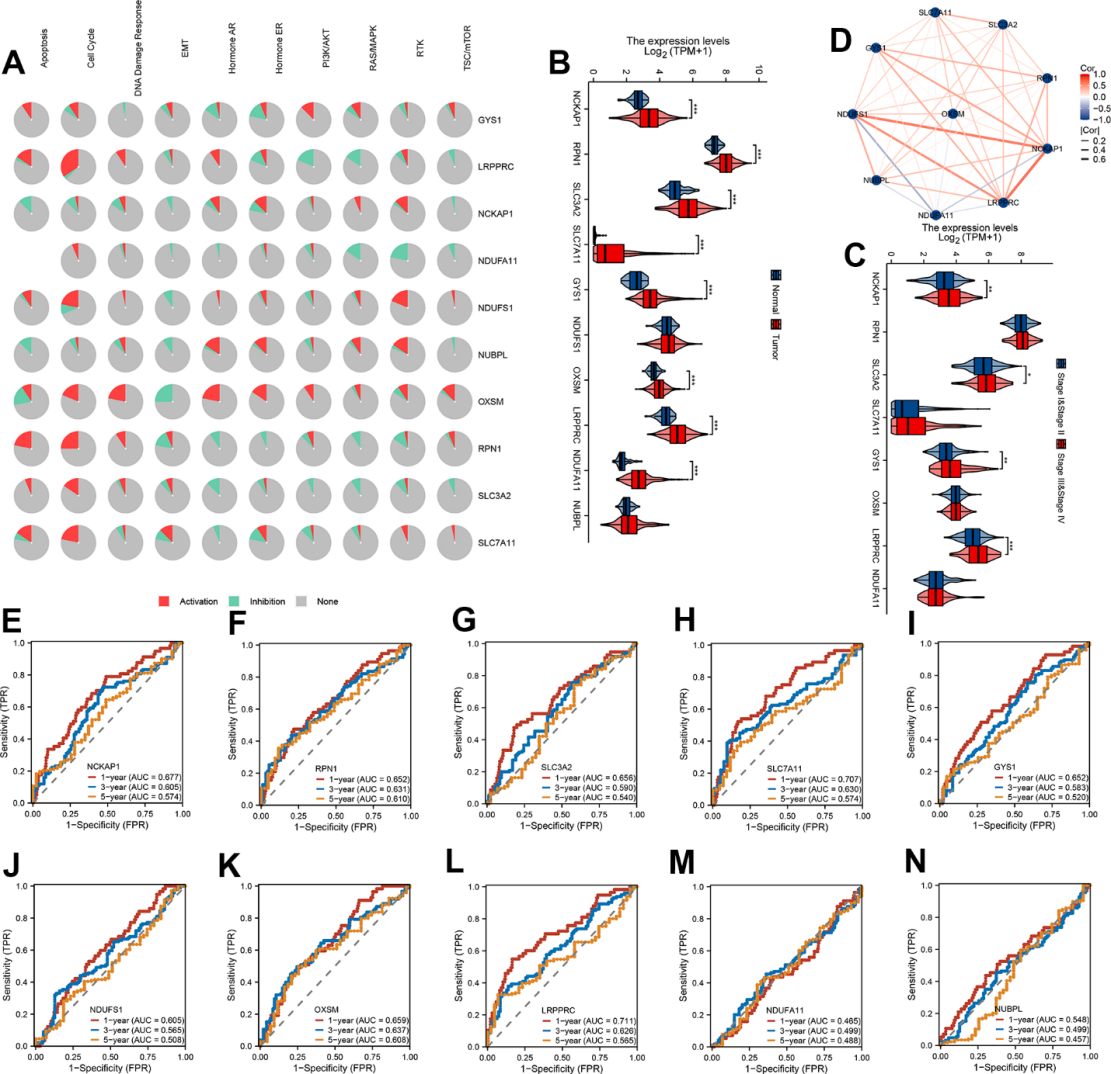
**mRNA expression levels and predictive efficiency of 10 DRG in TCGA-LIHC cohort.** (**A**) The relationship between 10 DRG and tumor signaling pathways. (**B**) The expression distribution of 10 DRG between tumor and normal. (**C**) The expression distribution of 10 DRG between stage III & stage IV than stage I & stage II. (**D**) Correlation map of 10 DRG. (**E**–**N**) ROC analysis showed the predict performance 10 DRG.

To confirm the biological function of 10 DRG in HCC, we first calculated the cancer major pathway score and then the correlation of 10 DRG with the cancer major pathway score. We found that 10 DRG mainly affects cancer development through the cell cycle pathway (Figure 1A). Next, the expression of 8/10 DRG were highly expressed in HCC except NUBPL and NDUFS1 (Figure 1B). Meanwhile, the expression of NCKAP1, SLC3A2, GYS1, LRPPRC were highly expressed in stage III and stage IV (Figure 1C). We also found that 10 DRG were closely positive correlation except NDUFA11 (Figure 1D). Next, to assess the accuracy of 10 DRG in predicting the prognosis of HCC, we plotted the ROC curves. The 1-year, 3-year, and 5-year AUC of NCKAP1 was 0.677, 0.605, 0.574, RPN1 was 0.652, 0.631, 0.610, SLC3A2 was 0.656, 0.590, 0.540, SLC7A11 was 0.707, 0.630, 0.574, GYS1 was 0.652, 0.583, 0.520, NDUFS1 was 0.605, 0.565, 0.508, OXSM was 0.659, 0.637, 0.608, LRPPRC was 0.711, 0.626, 0.565, NDUFA11 was 0.465, 0.499, 0.488, NUBPL was 0.548, 0.499, 0.457. The 10 DRG exhibited a low AUC area, suggesting that these gene had a worse overall performance (Figure 1E–1N). Therefore, there is an urgent need to develop a more effective model to predict the survival of HCC.

### SLC7A11 and LRPPRC can be used as an independent prognosis factor in HCC in TCGA-LIHC cohort

**Figure 2:**
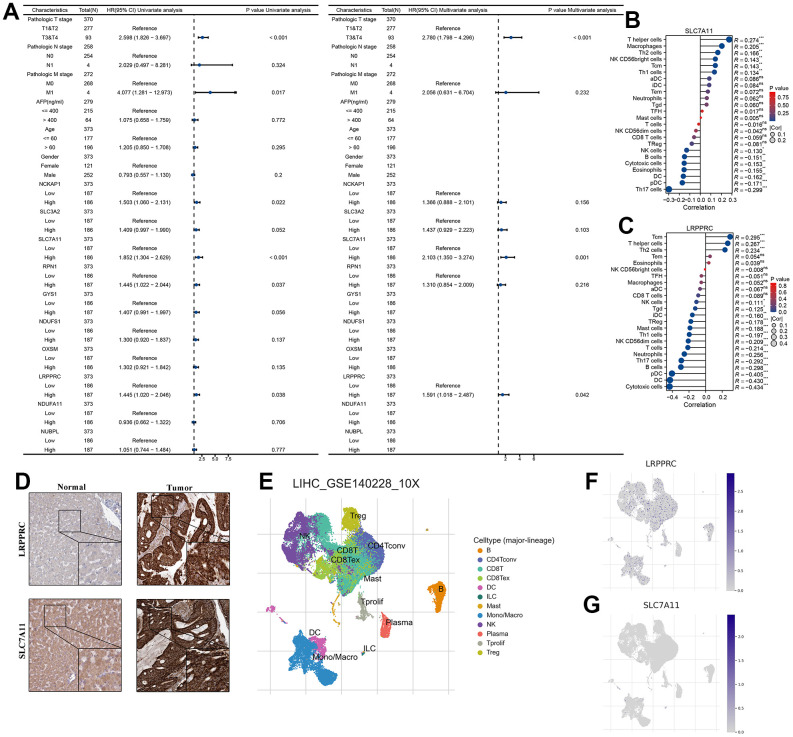
**SLC7A11 and LRPPRC can be used as an independent prognosis factor in HCC in TCGA-LIHC cohort.** (**A**) The univariate and multivariate Cox regression analysis between 10 DRG and overall survival. (**B**) The relationship between SLC7A11 and immune infiltration. (**C**) The relationship between LRPPRC and immune infiltration. (**D**) Immunohistochemistry images of SLC7A11 and LRPPRC expression in normal tissues, and HCC tissue. (**E**) T-distributed stochastic neighbor embedding plot of all the single cells, with each color coded for immune cell types. (**F**, **G**) The expression distribution of SLC7A11 and LRPPRC in immune cell types.

To assess whether 10 DRG, age, gender, TNM stage, and AFP were independent prognostic factors for HCC patients, our forest plot results obtained by univariate and multivariate COX regression analysis showed that both T stage and SLC7A11 and LRPPRC were independent prognostic factors for patients with HCC (P<0.05) (Figure 2A). Correlation analysis of immune cell subpopulations in ssGSEA showed that SLC7A11 expression levels were positively correlated with T helper cells, macrophages, Th2 cells, NK CD56bright cells, Tcm, Th1 cells and negatively correlated with NK cells, B cells, cytotoxic cells, eosinophils, DC, pDC, and Th17 cells. LRPPRC expression levels were positively correlated with Tcm, T helper cells, and Th2 cells and negatively correlated with other cells (Figure 2B, 2C). Further immunohistochemistry showed that SLC7A11 and LRPPRC were higher in cancer tissues than in normal tissues (Figure 2D). Next, our single-cell dataset was validated for SLC7A11 and LRPPRC expression. Based on the GSE140228 dataset, 62,530 cells were identified. Further clustering analysis resulted in surface these cells can be classified into 12 cell types (B, CD4Tconv, CD8T, CD8Tex, DC, ILC, Mast, Mono/Macro, NK, Plasma, Tprolif, and Treg) (Figure 2E). Among them, LRPPRC is widely released in various immune cells and SLC7A11 is mainly released in DC, ILC cells (Figure 2F, 2G).

### Molecular subtypes based on 10 DRG in the meta cohort

**Figure 3:**
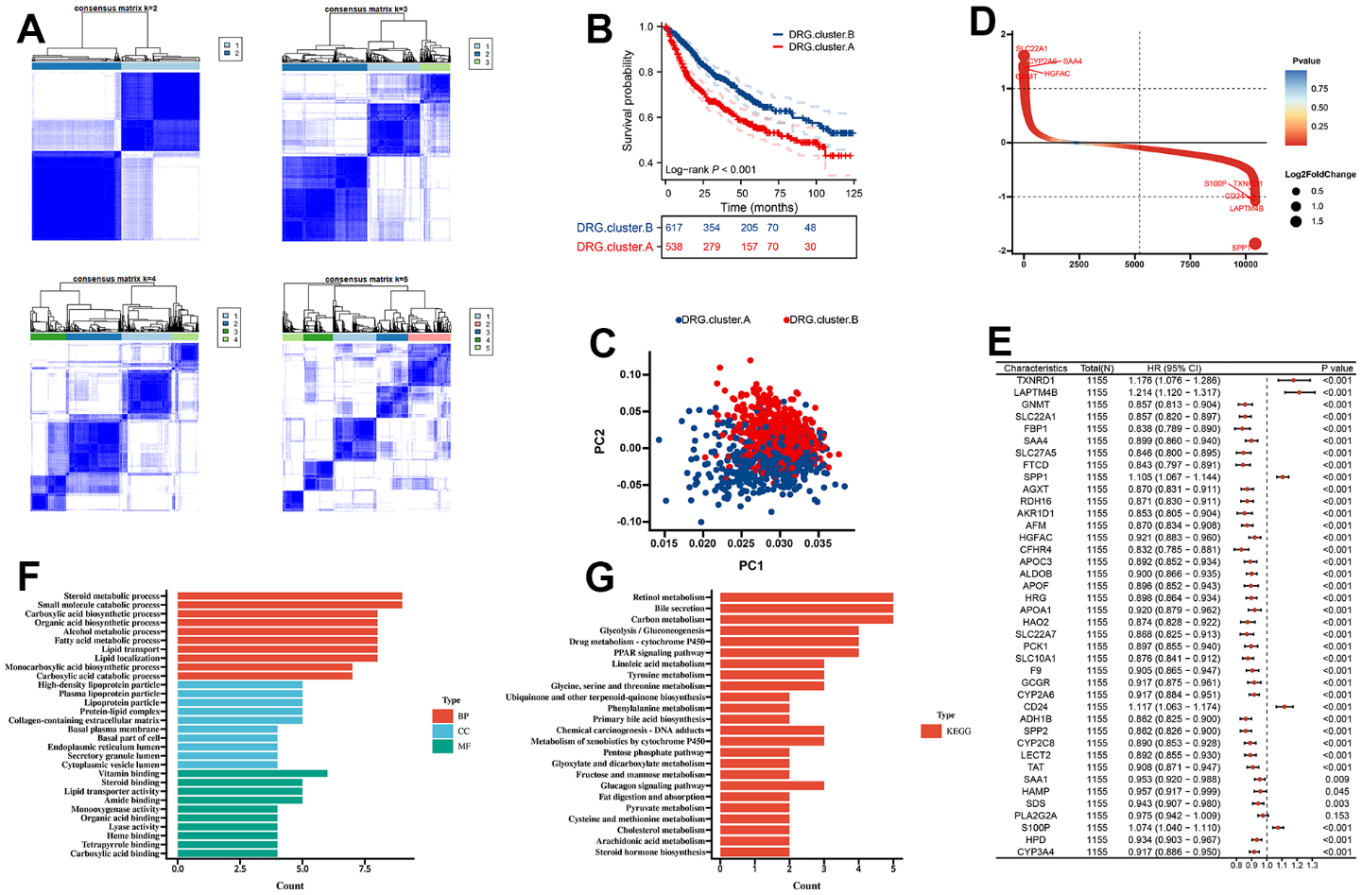
**Molecular subtypes based on 10 DRG in the meta cohort.** (**A**) Unsupervised consensus clustering based on 10 DRG for 1155 HCC patients in a meta cohort (GSE14520, GSE36376, GSE76427, LIRI-JP, and TCGA-LIHC). (**B**) Kaplan-Meier curve showed a significant difference between the 2 DRG.clusters. (**C**) PCA analysis between 2 DRG.clusters. (**D**) The different genes between the 2 DRG.clusters. (**E**) The univariate Cox regression analysis between 40 DEGs and overall survival. (**F**) GO enrichment analysis, (**G**) KEGG enrichment analysis for the DEGs and prognosis genes between the 2 DRG.clusters.

First, we merge TCGA-LIHC, LIRI-JP, GSE14520, GSE36376, and GSE76427 into a meta cohort. The consensus CDF curve and the change in area under CDF delta area curve showed that, for consensus matrix k=2, 9 DRG related expression-based classification had relatively stable clustering results (Figure 3A). We figured out that the OS time of the DRG.cluster.B had better prognosis compared with DRG.cluster.A (Figure 3B). Two clusters such as DRG.cluster.A and DRG.cluster.B were clearly identified (Figure 3C). A total of 40 mRNAs with significant differences between DRG.cluster.A and DRG.cluster.B HCC subtypes (|log2FC|>1; adj. p<0.05) (Figure 3D and Supplementary Table 1). By univariate COX regression analysis, 39 out of 40 genes were significantly associated with survival prognosis (Figure 3E). These genes were mainly enriched in metabolism-related signaling pathways (retinol metabolism, carbon metabolism, linoleic acid metabolism, tyrosine metabolism) (Figure 3F, 3G and Supplementary Tables 2, 3).

### TME between the 2 DRG.cluster

**Figure 4:**
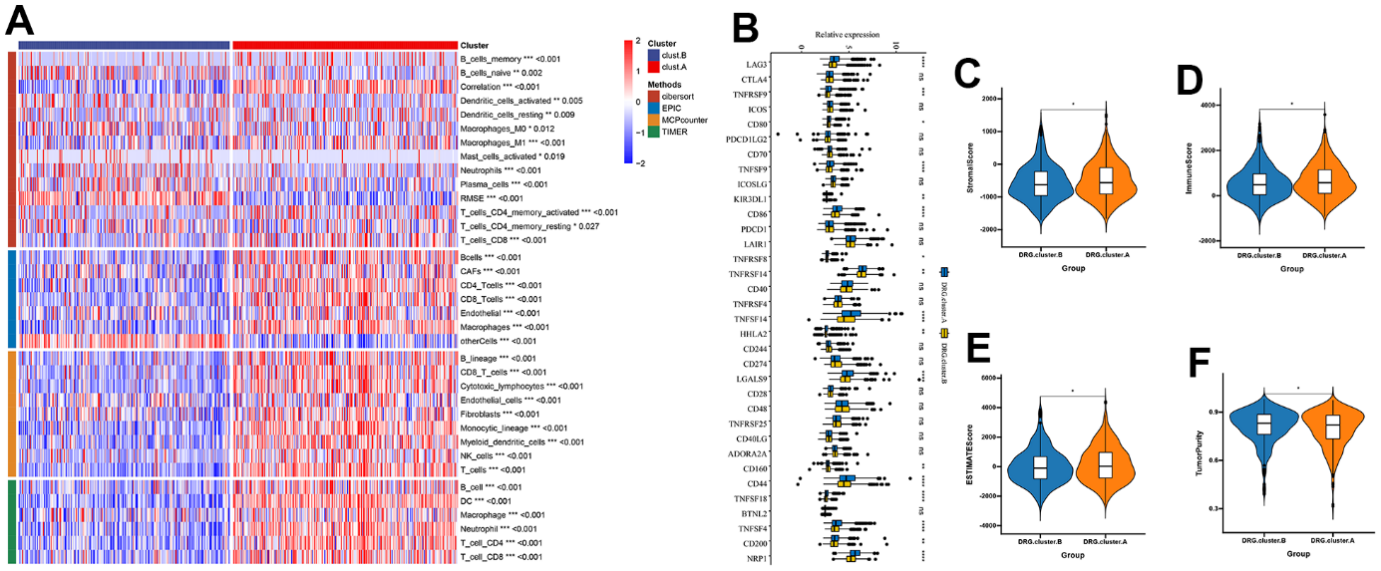
**TME between the 2 DRG.cluster.** (**A**) The relationship between the 2 DRG.clusters and TME. (**B**) The correlation of immune checkpoint condition in 2 DRG.clusters. (**C**–**F**) The ESTIMATE score, stromal score, immune score, and tumor immunity levels in 2 DRG.clusters.

Next, we further explored the immunological role of DRG.cluster in HCC. The results suggested that the infiltration level of most immune cells was significantly lower in DRG.cluster.B group (Figure 4A). In this analysis, we also evaluated some representative targets, and found that immune checkpoint were highly expressed in DRG.cluster.A group (Figure 4B and Supplementary Table 4). The ESTIMATE results showed that the stromal score, immune score and ESTIMATE score in DRG.cluster.A group were much higher than those in DRG.cluster.B group (Figure 4C–4F and Supplementary Table 5).

### Identification of hub genes between the 2 DRG.cluster

**Figure 5:**
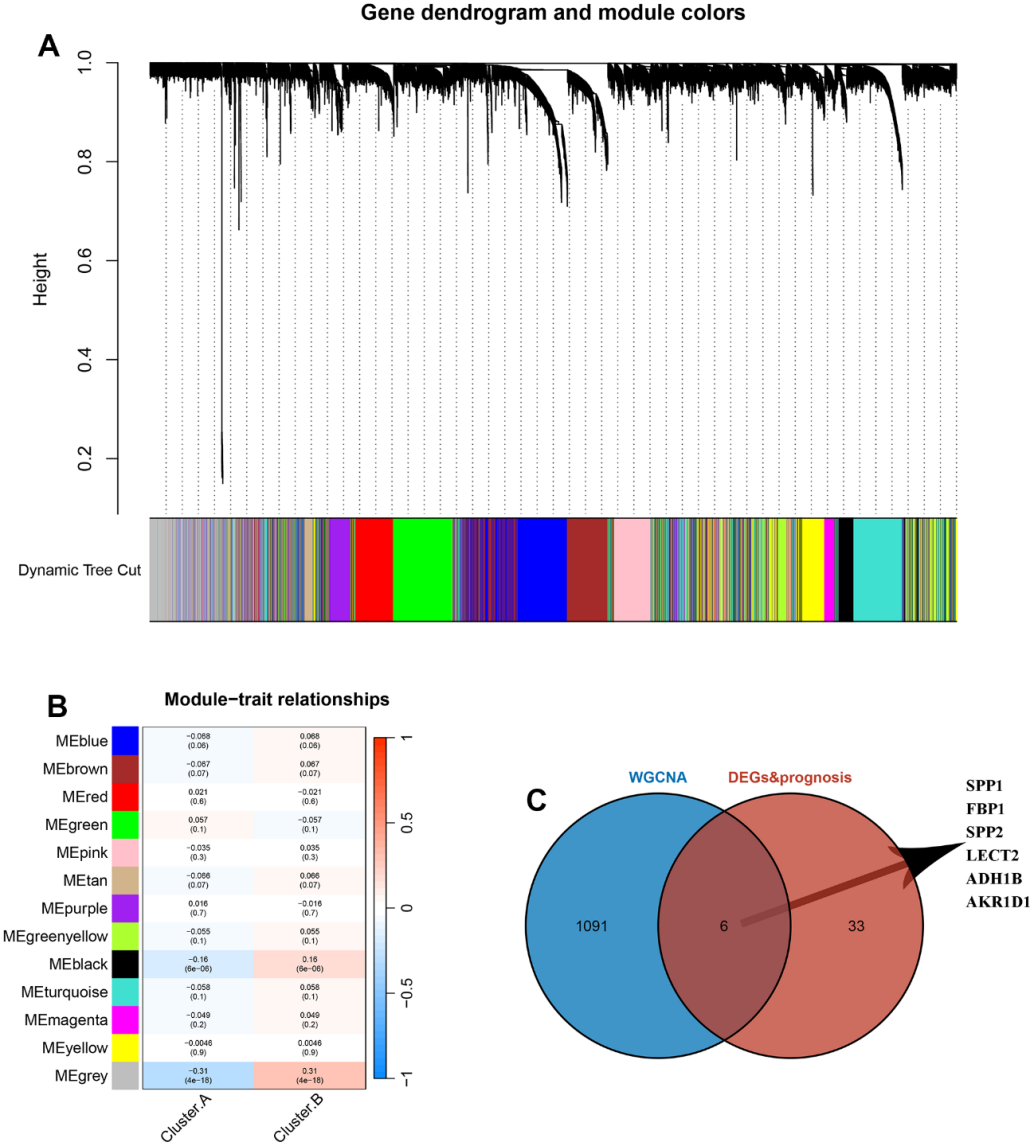
**Identification of hub genes between the 2 DRG.cluster.** (**A**) Co-expression network module clustering dendrogram, different colors represent different clusters. (**B**) Heat map of correlations between gene modules and clinical features of HCC, with red representing positive correlations and blue the opposite. (**C**) Venn diagram showing common genes between WGCNA and DEGs and prognosis.

The sample clustering tree was constructed based on dynamic hybrid cuts using scale-free networks and topological overlap. Based on the scale-free topology criterion, the optimal soft threshold β=4 was determined based on the fit index and the average degree of network connectivity (Supplementary Figure 1). Based on the optimal soft threshold, the gene modules were divided, the cut height was set to 25%, and a total of 13 modules were obtained, and the module cluster tree was drawn (Figure 5A). The correlation analysis between the construct modules and clinical characteristics was performed and heat maps were drawn, and the grey module correlated most closely with HCC (r=0.31, P=4e-18) (Figure 5B). Next, the genes in the grey module were extracted, and a total of 1097 genes were obtained. A total of 6 overlapping genes were obtained by intersecting the genes in the grey module with DEGs and prognosis through the Venn diagram. These 6 genes were defined as candidate pivotal genes in this study (Figure 5C).

### DRG.score model was constructed based on 6 hub genes

**Figure 6:**
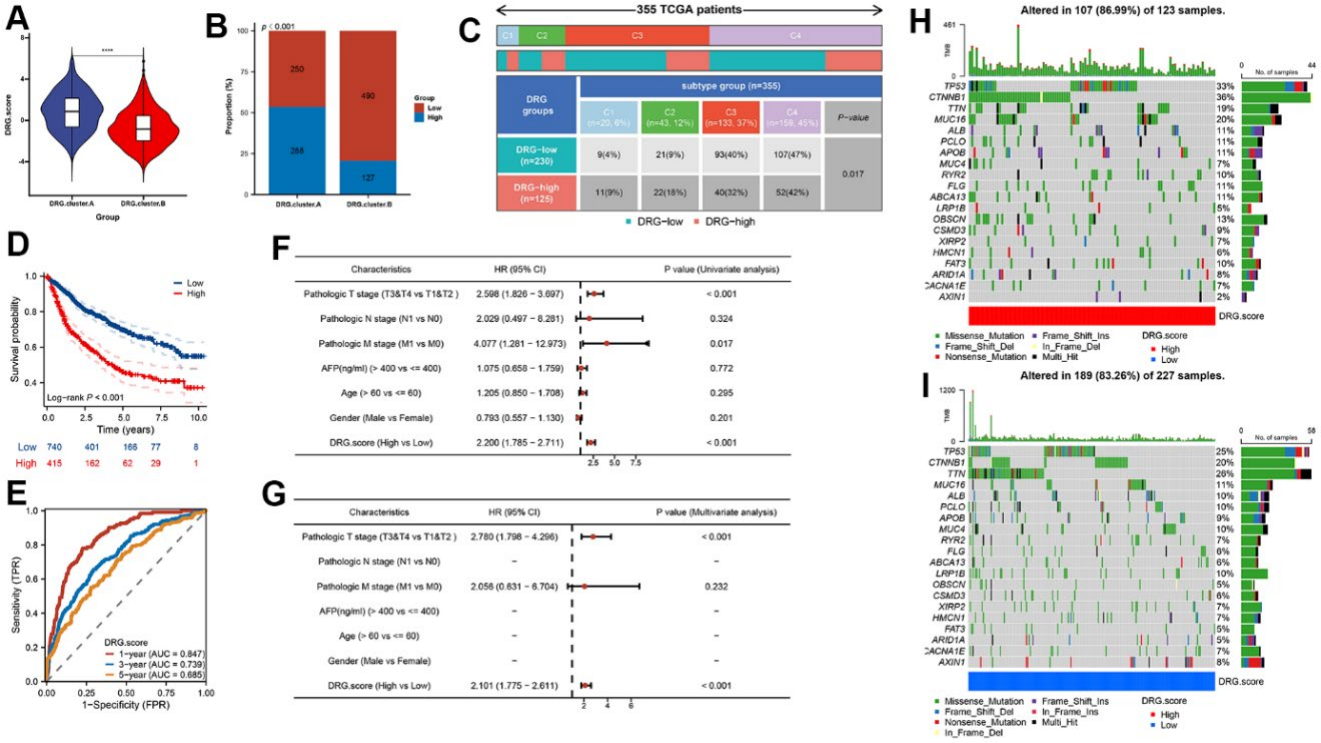
**DRG.score model was constructed based on 6 hub genes.** (**A**) Differences in DRG.score among 2 DRG.clusters. (**B**) The number of high and low DRG.score patients in 2 DEG.clusters groups. (**C**) Differences in DRG.score between immune subtypes. (**D**) Kaplan-Meier curves for high and low DRG.score groups. (**E**) The predictive value of DRG.score. (**F**, **G**) The univariate and multivariate Cox regression analysis between DRG.score and overall survival. (**H**, **I**) The waterfall plot depicted the differences in frequently mutated genes of hepatocellular carcinoma among high and low DRG.score groups.

In order to predict the prognosis of HCC patients more accurately, we built DRG.score based on 6 hub gene (Supplementary Table 6). According to the DRG.score distribution, the 2 DRG.cluster group had significant differences. The results suggested that the DRG.score in DRG.cluster.B group were much lower than those in DRG.cluster.A group (Figure 6A), and DRG.cluster.A group had much more high DRG.score patients (Figure 6B). TCGA-based molecular subtypes also showed that the DRG.scores were different for different subtypes (Figure 6C). The OS time curve showed that lower DRG.score subgroup had longer survival time (Figure 6D). The 1-year, 3-year and 5-year AUC of DRG.score was 0.847, 0.739, 0.685 (Figure 6E). The DRG.score exhibited a high AUC area, suggesting that DRG.score had a good overall performance. Meanwhile, univariate and multivariate Cox regression analysis show that DRG.score can be used as an independent factor to predict the prognosis in HCC (Figure 6F, 6G). Also, a lower mutation was discovered in the low DRG.score group (86.99% vs 83.26%) (Figure 6H, 6I). The above results show that the DRG.score can better predict the risk of prognosis.

### TME between the DRG.score groups

**Figure 7:**
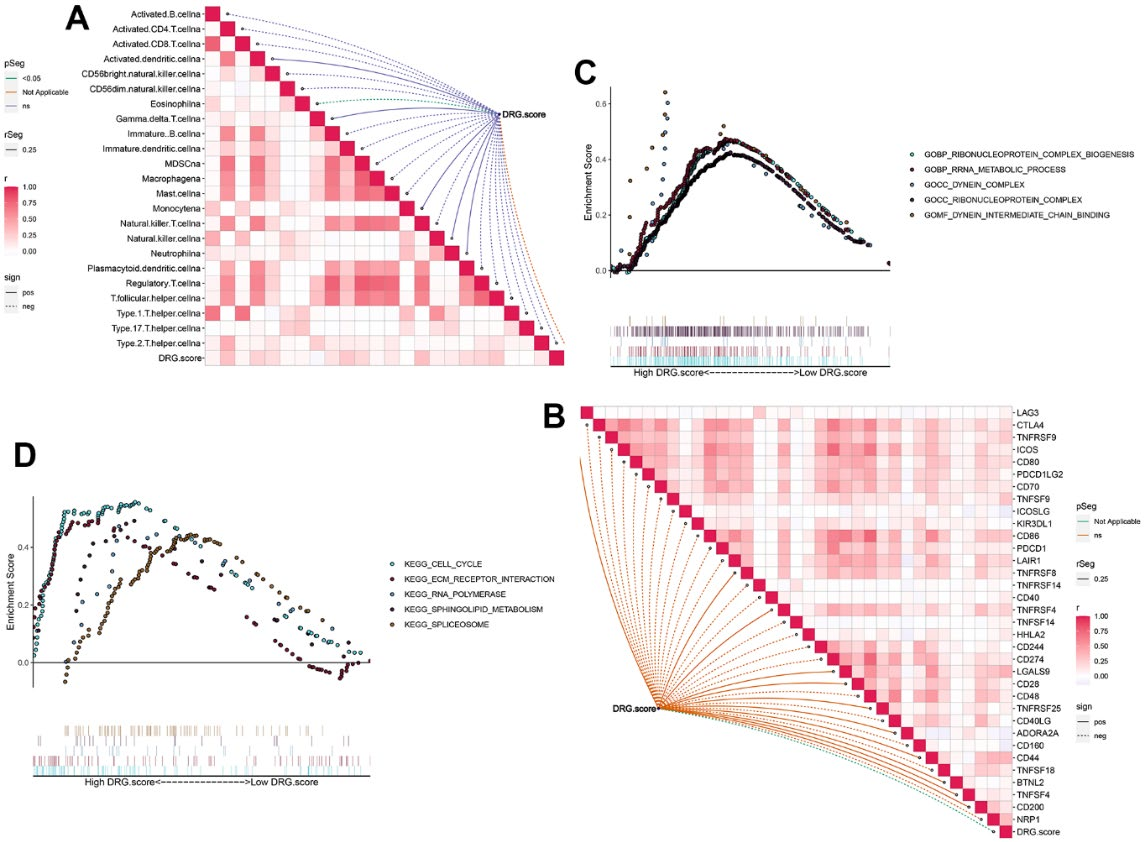
**TME between the high and low DRG.score groups.** (**A**) The correlation between DRG.score and different immune cells. (**B**) The correlation between DRG.score and different immune checkpoint. (**C**) GSEA GO identified high and low DRG.score groups related signaling pathways in HCC. (**D**) GSEA KEGG identified high and low DRG.score groups related signaling pathways in HCC.

Next, the results figured out that the positive correlation between these immune cells and DRG.score (Figure 7A). The infiltration level of immune cells was significantly lower in low DRG.score group (Supplementary Figure 2). We also found the positive correlations between these immune checkpoints and DRG.score were intricate (Figure7B). In order to examine the underlying biological processes in the high and low DRG.score groups, we performed a GSEA analysis. The results showed that tumor-related pathways (cell cycle, ECM receptor interaction) were enriched in the high DRG.score group (Figure 4C, 4D). These results suggested that high DRG.score may have a significant impact in tumor development.

### DRG.score is a robust prognosis factor in HCC

**Figure 8:**
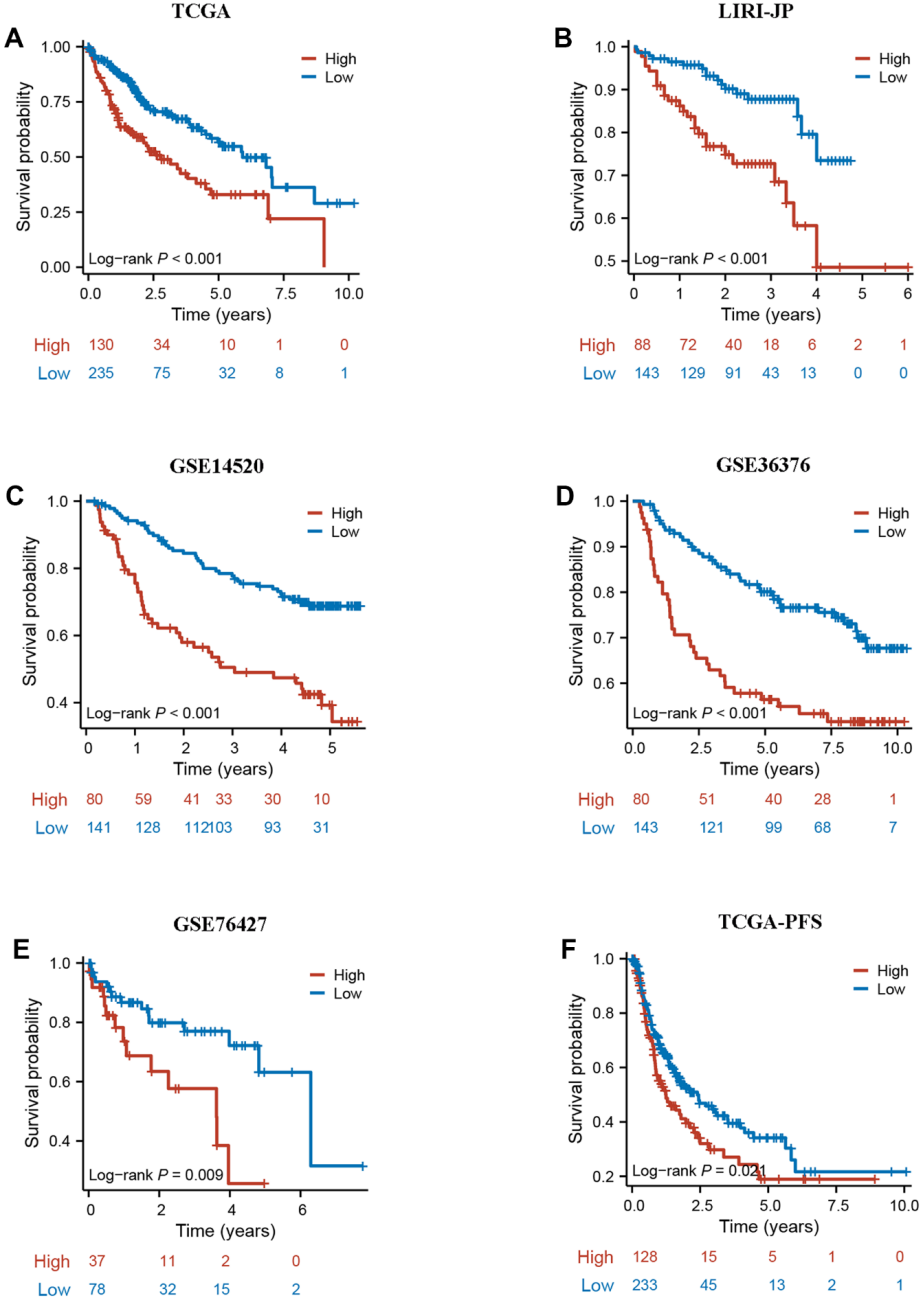
**External validation of DRG.score.** (**A**–**E**) The Kaplan-Meier curves analysis between high and low DRG.score groups and overall survival in GSE14520, GSE36376, GSE76427, LIRI-JP, and TCGA-LIHC. (**F**) The Kaplan-Meier curves analysis between high and low DRG.score groups and progression free survival in G TCGA-LIHC.

To further validate the robustness of DRG.score, the prognostic implication of DRG.score was examined in multiple independent datasets. In both patient sets, patients in the high DRG.score group had a worse OS than in the low DRG.score group in TCGA-LIHC, LIRI-JP, GSE14520, GSE36376, and GSE76427 (Figure 8A–8E). Meanwhile, patients in the high DRG.score group had a worse progression free survival than in the low DRG.score group in TCGA-LIHC (Figure 8F).

### Evaluation of the immunotherapy response between the DRG.score groups

**Figure 9:**
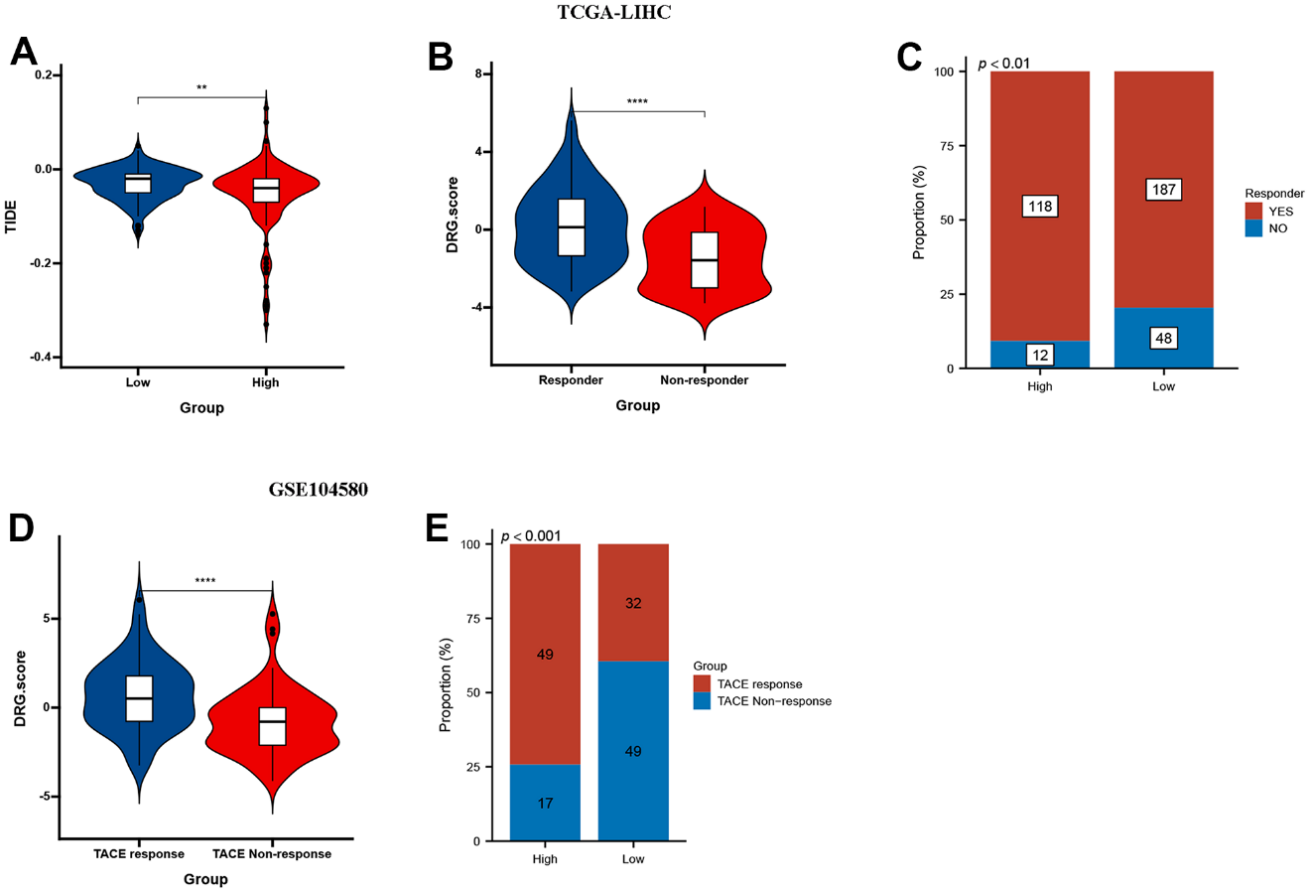
**DRG.score in the role of anti-PD-1/L1 immunotherapy.** (**A**) Differences in TIDE among high and low DRG.score groups. (**B**) Differences in DRG.score among non-response and response groups. (**C**) The proportion of non-response and response patients in low or high DRG.score groups. (**D**) Differences in DRG.score among TACE non-response and TACE response groups. (**E**) The proportion of TACE non-response and TACE response patients in low or high DRG.score groups.

The results showed that low DRG.score group had a higher TIDE score compared with high DRG.score group (Figure 9A). Higher TIDE scores indicate a greater likelihood of immune evasion, suggesting that patients may not benefit from immune checkpoint inhibitor therapy. Meanwhile, responder group had a higher DRG.score than non-responder group (Figure 9B), and high DRG.score group had a higher responder patients compared with low DRG.score group (Figure 9C). Moreover, a higher DRG.score was found in the TACE response group than in the TACE non-response group, and high DRG.score group had a higher TACE responder patients compared with low DRG.score group (Figure 9D, 9E). These results suggested that patients with higher DRG.score demonstrated significant immunotherapy therapeutic advantages and TACE clinical benefits.

## DISCUSSION

HCC remains an important public health safety issue worldwide, and the problem of tumor heterogeneity greatly constrains precision oncology treatment. One of the characteristics of tumor cells is resistance to normal death, which is the basis of the origin and development of cancer, so the inability of cells to self-kill is thought to be related to the growth and metastasis of cancer [26]. Disulfidptosis is similar to other normal cell death modes, ferroptosis helps gemcitabine inhibit resistance to pancreatic cancer, and pyroptosis affects all stages of tumor carcinogenesis [27]. Therefore, we believe that DRG are valuable in determining the occurrence and prognosis of HCC.

We focused on 10 DRG, constructed expression differential genes and prognostic differential genes related to disulfidptosis based on HCC expression and survival data in TCGA database. The SLC7A11 gene is located on human chromosome 4 and contains 14 exons. This gene is responsible for the uptake of extracellular cystine into the cell and the exchange of glutamate out of the cell in a 1:1 ratio, promoting the synthesis of the intracellular biological antioxidant glutathione (GSH) and protecting cell survival, and plays a key role in maintaining the balance of intra- and extracellular GSH [28, 29]. It has been demonstrated that in most tumors, the SLC7A11 coding code protein, through its specific biological properties, alters the microenvironment of tumor growth and thus promote tumor growth [30, 31]. Guo et al. found that the SLC7A11 gene increased the expression level of reactive oxygen species (ROS) in HCC cells and affected the growth of tumor cells. It was found that upregulation of SLC7A11 gene expression could activate AP-1 transcription factor, which could affect tumor uptake and metabolism of calcium ions and accelerate its cell cycle to promote tumor growth and proliferation. Additionally, the study found that SLC7A11 gene is usually elevated in HCC tissues, and is associated with a worse prognosis of hepatocellular carcinoma patients, and artificial damage to SLC7A11 inhibit the growth and appreciation of hepatocellular carcinoma cells, and SLC7A11 dysfunction has also been shown to increase the intracellular reactive oxygen species level, which in turn leads to autophagic cell death in hepatocellular carcinoma cells [32, 33]. LRPPRC protein is a multifunctional protein that regulates energy metabolism, participates in the maturation of nuclear-encoded mRNAs, and regulates signal transduction pathways. It was found that overexpression of LRPPRC gene was detected in various human malignancies, and overexpression of LRPPRC gene was strongly associated with poor prognosis of tumor patients [34, 35]. LRPPRC gene silencing significantly inhibited the growth and invasion of tumor cells, induced apoptosis, and reduced their drug resistance. The anti-apoptotic effect of LRPPRC was enhanced by a significant decrease in cytochrome C oxidase activity in hepatocellular carcinoma cells with reduced expression of LRPPRC. LRPPRC enters the mitochondrial oxidative metabolism by delaying apoptosis of hepatocellular carcinoma cells [36]. However, subsequent ROC curve analysis confirmed that SLC7A11 and LRPPRC did not show good predictive power, and this limits their predictive power as markers.

In order to obtain more effective markers to predict the prognosis of HCC, we first divided HCC into two molecular subtypes, among which the cluster.B had better prognosis and cluster.A had the worst prognosis. For immune subtype classification, we figured out that there was a significant difference in immune-related evaluation and clinical pathological signatures. These results demonstrated that distinguishing between DRG-based clusters provides a novel classification avenue for HCC. Next, 39 DEGs with prognosis was detected between the 2 clusters. Further, we used the WGCNA method to identify the most important genes in both groups and obtained a total of 1097 genes. Combining the genes obtained by these two methods, we identified the 6 hub genes, and DRG.score model was constructed based on 6 hub genes.

The TME is a complex heterogeneous environment composed of multiple cells. Infiltrating immune cells are an important component of the TME and are closely related to the efficacy of tumor immunotherapy and patient prognosis [37]. Tregs and macrophage M2 have been reported to be an important component of the TME with dual functions of immunosuppression, promoting tumor development [38]. In the present study, immunosuppressive cells such as Tregs, Th2 and macrophage M2 were more abundant in the high DRG.score group with poorer prognosis, while activated NK was significantly enriched in the low DRG.score group. The results suggest that the model is to some extent related to the immune landscape of the liver cancer microenvironment.

Traditional approaches include surgery, radiotherapy, chemotherapy, and targeted therapy. Although, the development of HCC-targeted therapy and other precision therapies have great breakthroughs have been achieved in recent years, the 5-year survival rate of patients has only increased from 7% to 15%. Lately, ICIs have been adopted in tumor therapy, transforming the tumor treatment’ paradigm. Tumor cells often escape cytotoxic T lymphocyte destruction by the upregulation of immune checkpoint ligands, such as PD-L1, which can inhibit lymphocyte activation by binding to the complementary receptor (PD-1) on cytotoxic T lymphocytes. In addition to CTLA4, PD-1, and LAG3, other immunosuppression-related genes, like IGFBP2 and LGALS1 are highly expressed in patients with glioma. Blocking the immune suppression-related genes’ expression can reshape the immunosuppressive TME. The immunosuppressive nature of the TME is a major cause of immunotherapy failure and chemoresistance in tumor patients. The varying response of each patient to immunotherapy and their heterogeneity make immunotherapy of tumors extremely difficult [39, 40]. In this study, DRG.score was significantly associated with ICIs therapeutic target genes, and significantly increased with increasing score. These findings suggest that this DRG.score model has the potential to predict the efficacy of ICIs. It has been shown that IFNγ released from CD8+ T cells downregulates the expression of two subunits (SLC3A2 and SLC7A11) of the glutamate-cystine reverse transporter system xc, which inhibits tumor cell cystine uptake and thus promotes lipid peroxidation in tumor cells [41]. These results suggest that the combination of induction of disulfidptosis in tumor cells and ICIs will hopefully provide a new perspective for tumor therapy.

Our study had several limitations. Critically, our study lacks experimental data and clinical validation, we need to verify our findings from patients or *in vitro* experimental in the subsequent studies to clarify the underlying mechanism of 6 signature genes in HCC. Secondly, we found DRG.score was also positively correlated with multiple immune features; however, we don’t know whether DRG.score dependent on the immune features in the association analysis with survival and immunotherapy efficacy.

In summary, this is a first study to analysis disulfidptosis-related genes in hepatocellular carcinoma, and we identified that SLC7A11 and LRPPRC could be used as independent prognostic factors for HCC. Meanwhile, the DRG-based classification and DRG.score model can facilitate the prediction and the selection of HCC individual and personalized immunotherapeutic.

## Supplementary Material

Supplementary Figures

Supplementary Table 1

Supplementary Table 2

Supplementary Table 3

Supplementary Table 4

Supplementary Table 5

Supplementary Table 6
