# Supplementary material for: Identification and characterization of a novel molecular classification based on disulfidptosis-related genes to predict prognosis and immunotherapy efficacy in hepatocellular carcinoma
Source: Aging (Albany NY). 2023 Jul 3;15(13):6135–51. doi: 10.18632/aging.204809 (PMC10373967; doi:10.18632/aging.204809)
Supplement: Supplementary Figures [file aging-15-204809-s001.pdf]

SUPPLEMENTARY FIGURES

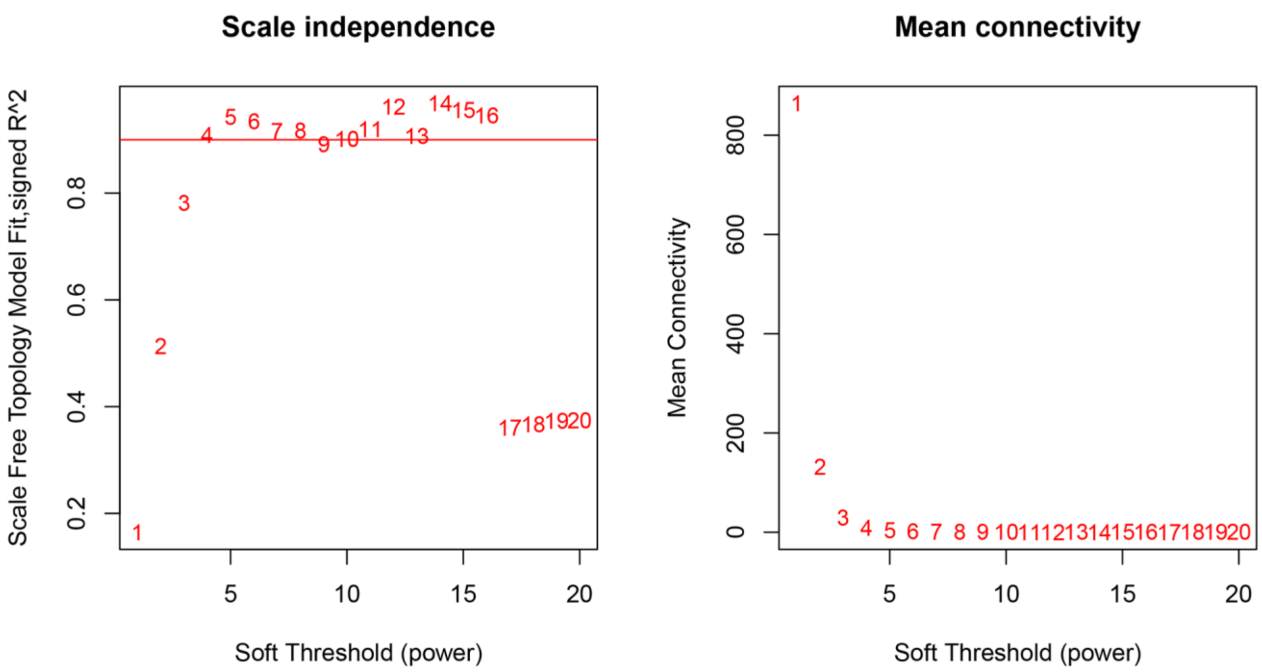

Supplementary Figure 1. Correspondence between the fit index and the soft threshold value, when the soft threshold value is 4, the fit index is >0.9 for the first time.

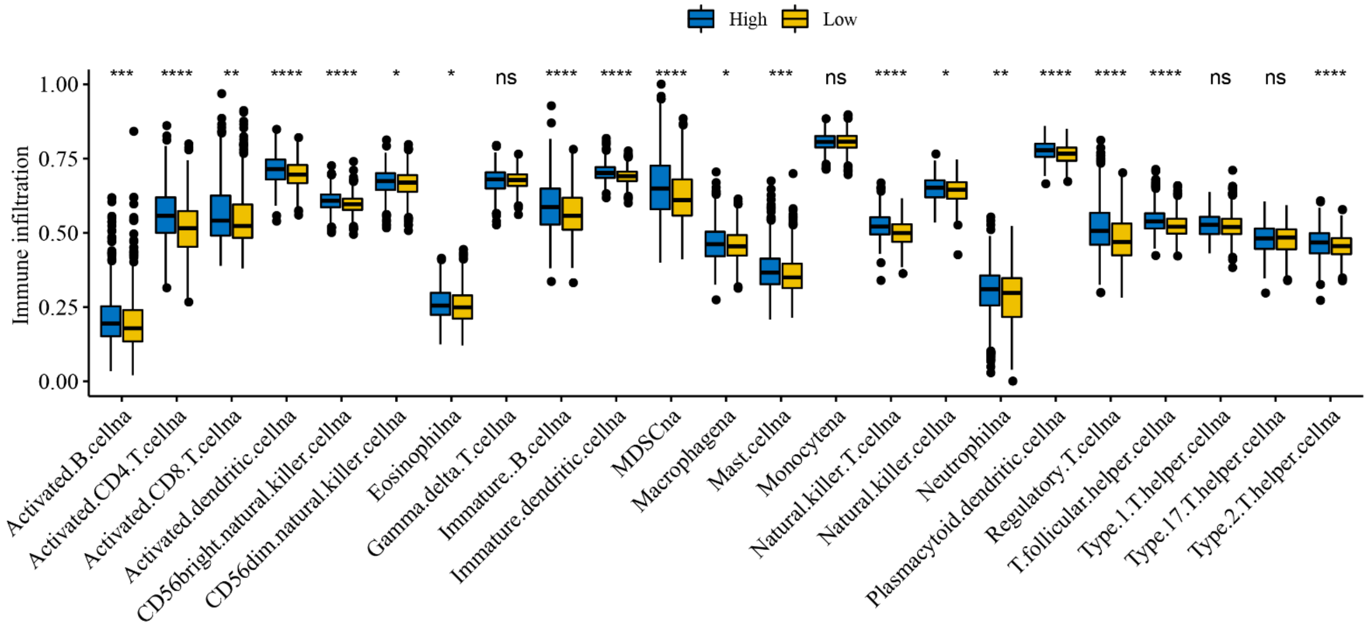

Supplementary Figure 2. The relationship between the high and low DRG.score groups and TME.
